# Supplementary material for: The DREAM Dataset: Supporting a data-driven study of autism spectrum disorder and robot enhanced therapy
Source: PLoS One. 2020 Aug 21;15(8):e0236939. doi: 10.1371/journal.pone.0236939 (PMC7444515; doi:10.1371/journal.pone.0236939)
Supplement: Attachment — Submitted filename: Rebutal letter.pdf [file pone.0236939.s001.pdf]

Rebuttal letter for PONE-D-20-08566:

The DREAM Dataset

Dear Prof Billeci, reviewers,

thank you for your decision letter and for the referee's comments concerning our manuscript entitled "The DREAM Dataset: Supporting a data-driven study of autism spectrum disorder and robot enhanced therapy".

We have read the referees' comments carefully and revised the manuscript on several grounds. Based on the referees' comments, we have made corrections which we hope meet with their approval. The reviewers' comments with our specific reply to every suggestion are listed as follows. Please also refer to the marked-up version of the manuscript, with all changes highlighted in blue, and to the cover letter for further details.

Reviewer	Comment	Response
1	Thank you for the opportunity to review this very interesting manuscript entitled "The DREAM Dataset: Supporting a data-driven study of autism spectrum disorder and robot enhanced therapy" which has been submitted for consideration in the Plos One. Robot Enhanced Therapy attracted great attention. I agree the importance of Robot Enhanced Therapy and dataset supporting further data-driven studies toward improved therapy methods as well as better understanding of ASD in general. However, I could not find any result in this article. In addition Discussion is very short.	Thank you for these encouraging comments. We have discussed the question of results extensively in the author team and concluded that this article makes a sharper contribution by focusing on the presentation of the dataset itself, without covering a particular aspect of analysis. Following a suggestion from reviewer 3, we have however added some descriptive statistics of the dataset in Sec. 4 and Fig. 3. You are right that the discussion is short and while we have modified it slightly based on recommendations from other reviewers, we've decided not to extend it significantly.
	The MS describes a database/set corresponding to a study involving a robot (robot enhanced therapy) in children with ASD. Authors have recorded many features during 3000 sessions and they offer a data visualizer that is very welcome. Given the unique dataset they are describing, authors should be congratulated for such a commitment.	Thank you.
2	However they are many imprecisions and writing issues that make the manuscript inadequate for publication. Authors need to work on it a bit more (It looks like a conference paper that we often have in the field). The intro does not stand as it is. It should be reorganized and some points need to be added or modified. I suggest:	We have made our best to address all suggestions and also made other adjustments of the article, details below.
2	- start by ASD treatment principles (see Narzisi et al.	Large parts of the introduction have been

	2015).	rewritten including these suggestions, but also suggestions from other reviewers. Additions are marked in blue and found on page 2 and 3.
2	- Don’t start by medication!! It is not the treatment of autism.	
2	- The claim that medication has strong evidence is wrong! The statements on medication are useless.	
2	- Then be more specific with ABA (because) it inspired your robot enhanced therapy.	
2	- Add a brief paragraph on robot and ASD (they are several recent reviews).	
2	- Then present RET and indicate in the intro the exact design (randomized?, duration?)	
2	- Page 2 correct form for from	Done
2	The clinical evaluation:	
2	- This section should be renamed “clinical evaluation and protocol”	Done
2	- The most important revision should be done when authors describe the setting in this section. To me, it should be a separate section called “sensors and setting”.	We appreciate this suggestion and have added a new section for sensing and setting, 3.1
2	- In this new section, the authors should detail more how the robot is used (at least grossly as I understand that details would be available in the clinical paper). Also they should offer a table with the extracted features and made available in the dataset (and the corresponding algorithm they used). Also they should indicate whether some raw data are availables.	A table (Table 1) is now included specifying each extracted sensor primitive and corresponding method used. Raw data can not be released, which is stated in the introduction and has now also been clarified in Sec. 4
2	3. The section open data set needs a 4.1 “dataset variables”. Also the 4.2 “licence” seems to be a very general statement found on websites! Please if you give a note be more specific in the case of Dream data set. No need of general legal statements!	The formulation of the licence section is standardized because this is what the selected CC-licence states. We've added a clarification of exactly what the licence covers and not.
2	4. The discussion is minimalist and why not, I am OK. But please edit the section as there are at least 2 sentences that I did not understood.	Done
2	Also, please cite more recent reviews instead of [11, 14].	Several relevant references, including the review by Pennisi et al. 2016, has been included and referenced in the introduction.
2	Finally, the statement very general that the data set will offer opportunity to develop new screening method makes no sense to me. You don’t have typical developing controls!	While the present dataset lacks an explicit control group in the form of typically developed children, the children involved in the study varied in autism severity both in terms of total ADOS scores and symptom type. Thus, the data could be used to investigate behavioural cues that support predictors for a specific ASD score, and in

		that way provide input to screening. That said, we also see the drawback of not having a control with typically developed children and have therefore changed the statement to the following: ... Such patterns may guide further clinical studies by providing new insights into how to appropriately select between RET and traditional ABA therapies or constitute input to new therapeutic methods.
3	This is really impressive work and I commend the authors on what they have achieved in this project. Such a through dataset is a valuable contribution to the field and will undoubtedly be useful to other researchers.<br><br>My only suggestion is that the authors provide more details on the duration of the therapy sessions in their overview. Currently the authors say that sessions lasted anywhere between a few minutes and 40 minutes. It would be useful to have a graph batching the sessions and providing an indication the average session might last and also if theses session durations increased or decreased over the course of the study.<br><br>Overall great work!	Thank you for these encouraging comments!<br><br>The session length varied as a result of script length and child performance, but was also dependent on how sessions/sittings a specific intervention was divided into. After some consideration we therefore found it more suitable to report the total intervention length (i.e., the sum of all sessions in a specific intervention). This is now clarified in the beginning of Sec. 4. We also took your suggestion and added a new figure (Fig 3) displaying the intervention durations over the complete protocol.
4	About the content of the dataset itself: Page 8, in the list of included data, I would replace 'date' by 'time', so that it is clear that not only the day is specified. Shouldn't the success of the current trial also be indicated? This could help users of the dataset distinguish correct from incorrect movements, and maybe better understand cases of hesitation for instance. Moreover, the authors put an emphasis in the introduction on the different levels of difficulties/deficits of people within the ASD spectrum. Shouldn't the dataset provide information distinguishing these different levels?	The date has been replaced with time on page 8, as suggested. We also appreciated the suggestion to include difficulty level. We have therefore made an update to the dataset specification (Appendix A) and the complete dataset, now including a definition of task, with both targeted ability and difficulty. Unfortunately we can not release task performances (success on trial) at this stage since these are part of clinical results in the process of being published elsewhere.
4	Lines 19:23, the sentence is ill-formulated: '[5] labeled two interventions as well-established: individual, comprehensive Applied Behavior Analysis (ABA) and teacher-implemented, focused, ABA+ developmental social-pragmatic (DSP), an intervention that combines ABA and DSP strategies.' When I read the sentence, it seems to me that there are three interventions: ABA, DSP and ABA+. While actually there are ABA and ABA+DSP. And the way the sentence is written could suggest that DSP is an intervention that combines ABA and DSP. I suggest reformulating and separating the interventions with semicolons. Also, rather than following the way they are presented in the abstract of Smith & Iadarola 2015, it	The paragraph has been edited and this sentence rewritten.

	seems to me that it would be better to first list ABA and DSP, with a short explanation of what is the difference. And then explain that a combination of the two called ABA+DSP also exists. Then the authors could explain that Smith & Iadarola 2015 have emphasized their difference in efficacy. I am not sure it really useful to mention the distinction between focused and comprehensive, individual vs. teacher-implemented, since this is not further explained nor used in the present manuscript.	
4	Lines 33-34: 'an ABA protocol where a humanoid robot constitutes the interaction partner.' I think 'the' is not appropriate here. Because it suggests at first glance that the robot is the unique interaction partner. I would replace with 'an'. At the end of the paragraph, I think it would great to emphasis that in RET the goal is not to replace the human therapist by a robot, but instead to assist the therapist, the robot being only a mediator (of the therapy, as opposed to the therapist being a mediator of the interaction with the robot) or a tool.	Well spotted. We agree than "an" is a better term. Also, an introduction to RET as robots as mediators if interaction has been added to the beginning of Sec. 3, to give the clinical study more clarity in this regard
4	I think it is important to state that the robot's behavior is preprogrammed, not allowing any on-the-fly learning while interacting with the child. The strengths of doing so could be emphasized, such as stability, predictability, perhaps easier acceptability by children with ASD, and making sure that the behavior of the robot during the experiment is perfectly controlled. In contrast, it would interesting to mention that alternative studies enable the robot to learn on-the-fly while interacting with children with autism. The strengths and weaknesses of doing so could also be discussed in comparison with the present method. I think this would be very useful, first so that potential users of the dataset know clearly what was the robot's behavior and its abilities, and second to provide some insights to the community about the pros and cons of enabling robots to learn or not during RAT/RET.	This is a very relevant point, thank you for highlighting. We've now added a paragraph at the end of Sec. 3 on the topic: While sensor data was used to guide the robot's behavior on-line, responses were kept consistent throughout the study, i.e., the robot did not "learn" from previous interactions with the child. Instead, suitable task difficulty was achieved through the session scripts as described above, combined with supervised autonomy ensuring reliable robot behavior even in cases when the system failed to correctly assess the child's actions \cite{Esteban2017}. While this architecture could effectively be combined with robot learning \cite{Senft2016}, we here chose a static system in order to increase validity of the study.
4	Page 3/16, the authors should not forget to remove the last three sentences of footnote 2 before publication.	Done
4	One the one hand, the background section stresses the potential of this kind of dataset to contribute to the diagnosis of ASD. On the other hand, the introduction mentions only therapies, but not the use of robots in diagnosis. I think the objectives should be more clearly stated, and the potential contribution to therapy, diagnosis or both should be discussed. This of particular interest for the social robotics community which is currently wondering	Thanks for this comment, value for diagnosis, screening and improved understanding of ASD is added to the introduction.

	whether there is a potential for social robots to contribute to therapy only, or also to diagnosis.	
4	Line 98, ‘It focus specifically’ -> It focuses /or/ Its focus is.	Done
4	Figure 1 seems to suggest that no supervising human was involved in the SHT configuration. Could the authors confirm? Did the supervisor have an active role (like intervention), in addition to controlling the robot in case of problem, or only a passive role (monitoring)? In the former case, was it a problem not to have a supervisor during SHT? Was there a difference between RET and SHT in terms of interventions by the supervisor?	Clarification has been added in Sec. 3: The supervisor's role is to monitor the automatic interpretation of the child's behavior and to adjust the robot's responses if necessary. In the SHT condition, the supervisor is not present.
4	Figure 2 does not explicitly refer to any touchscreen between the child and the robot. The sandtray is actually not clearly visible in Figure 2, in contradiction with what is written (lines 164-166).	The statement has been removed
4	Line 170: ‘were employed’ -> was employed.	Done
4	Line 206, objected -> object.	Done
4	Lines 231-232: ‘state of the art sensor interpretation algorithms’. Could the authors more explicitly state which algorithms were used? I think it is important for people using the dataset to know.	Clarification has been added in Sec. 3
4	What happened when children only spent ‘a few minutes’ in a session? Did he/she complete only a single intervention script? Was the script completed? Was the data still included in the dataset?	Clarification has been added in Sec. 3: The length of each session varied from a few minutes up to 40 minutes as a result of script length and child performance.
4	Line 313, ‘screen shoot’ -> screenshot.	Thank you for these detailed comments, changes have been addressed.
4	Line 327, ‘be used studies’ -> be used by studies.	
4	Line 352, comprise -> comprises.	
